# From Initiative to Influence: The Impact of ICHR on Pakistan’s Health Research Ecosystem (2023–2025)

**DOI:** 10.12669/pjms.42.2.13233

**Published:** 2026-02

**Authors:** Naseer Ahmed, Nadia Anwar, Muhammad Rehman, Ghulam Rasool

**Affiliations:** 1 Naseer Ahmed Rehman College of Dentistry, Peshawar, Pakistan; 2 Nadia Anwar Rehman College of Dentistry, Peshawar, Pakistan; 3 Muhammad Rehman Rehman Medical Institute, Peshawar, Pakistan; 4 Ghulam Rasool Rehman College of Dentistry,Peshawar, Pakistan

**Keywords:** Academic conferences, Capacity building, Health research, International conference on health research, Research ecosystem

## Abstract

**Objective:**

In many low and middle-income countries, including Pakistan, health research often takes a backseat due to limited funding and collapsed infrastructure. Realizing this gap, Rehman Medical Institute, in collaboration with Rehman College of Dentistry, launched the International Conference on Health Research (ICHR) in 2023 to encourage a culture of evidence-based dialogue and capacity development. This study explores the evolution and impact of ICHR from 2023 to 2025, assessing trends in participation, skill development efforts, international alliances, and its influence on Pakistan’s academic landscape.

**Methodology:**

We conducted a descriptive retrospective study using data from official ICHR programs, registration records, workshop schedules, speaker lists, and institutional reports and feedback Forms. Post-conference feedback surveys provided insights into participant satisfaction, learning outcomes, and recommendations. Data were analyzed thematically and descriptively to identify trends and emergent patterns.

**Results:**

Participation rose by 233%, from 600 in 2023 to over 2,000 in 2025. Workshops expanded by 420%, growing from 10 to 52, and trained over 1,000 professionals in 2025 alone. Speaker diversity increased with representation from over 10 countries. Post ICHR-25, the Certificate in Health Research (CHR) program saw 200+ new enrollments in one month. Innovations such as hybrid formats, AI-integrated sessions, and student-led initiatives enhanced engagement and broadened impact.

**Conclusions:**

ICHR has emerged as a locally rooted yet globally relevant platform that promotes research training and dialogue. Its growth demonstrates how structured academic engagement can reshape institutional priorities and serve as a replicable model for academic conferencing and research capacity development across similar low- and middle-income countries.

## INTRODUCTION

The landscape of healthcare is rapidly changing, with new diseases emerging and technologies evolving at a pace that often surpasses policy and practice. Alongside these developments, the burden of non-communicable diseases and recurring global health emergencies has placed a renewed emphasis on the need for evidence-based practices and research.^1^,^2^ In this context, the need for evidence-based research has never been more urgent, especially in countries like Pakistan, where many decisions still rely on borrowed evidence from other health systems and external standards.^3^,^4^

Building research capacity is a slow and complex process. Many institutions lack formal training programs or sufficient funding, and healthcare professionals often view research as optional or out of reach.^5^ Rehman Medical Institute, in collaboration with Rehman College of Dentistry, took a pioneering step to address this issue and introduced the Certificate in Health Research (CHR,) a uniquely structured program designed to help doctors, dentists, nurses, and allied health workers develop essential research skills.

The success of the CHR program led to a broader institutional vision to have a platform that could bring together research-oriented professionals from across Pakistan and beyond for exchange of knowledge, collaboration, and innovation. This vision took shape as the International Conference on Health Research (ICHR) in 2023, launched by RMI-RCD. Initially conceived as a regional academic initiative, the original idea was to provide a dedicated platform for our own CHR graduates to present their work, engage with peers, and gain visibility in the wider research community. In just three years, ICHR has rapidly expanded into a national and international forum, attracting participants from diverse disciplines, institutions, and countries.

This paper critically examines the transformation of ICHR from 2023 to 2025, analyzing its thematic development, participation dynamics, internationalization, and broader impact on Pakistan’s research culture.

## METHODOLOGY

A descriptive retrospective study was conducted to evaluate the growth and impact of ICHR.. Data were compiled from official ICHR programs (2023–2025), registration databases, workshop schedules, speaker rosters, institutional reports from RMI, and post-conference participant feedback surveys. Quantitative metrics, including registration numbers and workshop count, were analyzed descriptively, while qualitative feedback was reviewed thematically to identify key trends related to participant satisfaction, perceived relevance, and areas of improvement. Comparative analysis across three years was used to assess progression and innovation. The analysis focused on four key areas:

1. Evolution in conference themes over time.
2. Increase in number of participants and institutional engagement.
3. Participation of international speakers and experts.
4. Workshops and innovations introduced each year.

We compared each year side by side to identify trends and improvements. While some conclusions are based on registrations, others take into account participants’ feedback, event quality, and ICHRs’ national and global recognition.

### Thematic Evolution:

Both local significance and global responsiveness can be seen in ICHR thematic arc;

- **ICHR-23:** Emerging Research Landscape: A Global Trend
- **ICHR-24:** Global Health in Transition Through Research: A Road Map to Future Realities
- **ICHR-25:** From Discovery to Impact: Strategies for Effective Implementation in Health Research.

In accordance with the international trends in academic conferencing, these evolving themes show a shift from communicating fundamental knowledge towards implementation science.^6^ Progressive theme refinement shows an improvement of institutional ambition and academic importance. The foundation was initially set by ICHR-23, which analyzed global patterns and encouraged participants to have a research-conscious mindset. By ICHR-24, a dialogue had begun to address the global health systemic shifts, urging researchers to critically engage in emerging healthcare paradigms. Finally, ICHR-25 represented a clear trend towards implementation science and a desire to provide knowledge but to also apply it meaningfully in practice and policy. This shift from producing knowledge to practical application displays how ICHR promotes the global movement for translational research and the advancement of health systems.

### Ethical Approval:

Ethical Research Committee Approval was obtained on 12 June 2025 Ref no: RCD-06-25-00256.

## RESULTS

### Participants’ growth over time:

**Fig.1:**
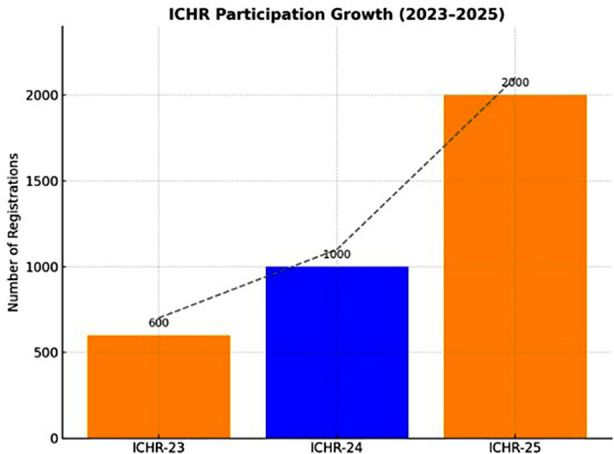
Participant Registrations ICHR-23 – ICHR-25.

The growing number of participants at ICHR is a clear indication of its developing influence. Participation in ICHR increased considerably between 2023 to 2025. There was a 66.7% increase in registrations from 600 in ICHR-23 to 1,000 in ICHR-24. As seen in Fig.1, registrations in ICHR-25 exceeded 2,000, a 100% increase from the year before. Besides the growing number of registrations, ICHR has developed into a uniquely inclusive platform that unites a diverse array of healthcare professionals. Participants included physicians, dentists, nurses, allied health professionals, public health specialists, biomedical scientists, physiotherapists, and medical editors, all engaged in meaningful dialogue on a single platform. This multidisciplinary setting fostered collaboration, promoted multidisciplinary learning, and produced an environment that was conducive to translating health research. A higher participation rate indicates enhanced engagement with academic discourse and collaborative inquiry.

This expansion is a result of growing academic curiosity, relevance across disciplines, and global recognition.^7^ Furthermore, it also represents the Rehman Medical Institute’s development of a long-term research culture. By consistent funding in research education initiatives like the CHR program, RMI has established an ecology that encourages sustained academic involvement in addition to drawing individuals from a variety of backgrounds. RMI’s deliberate emphasis on integrating research as a fundamental academic value is the direct cause of the noticeable excitement observed in ICHR engagement.

### Speaker diversity and internationalization:

**Fig.2:**
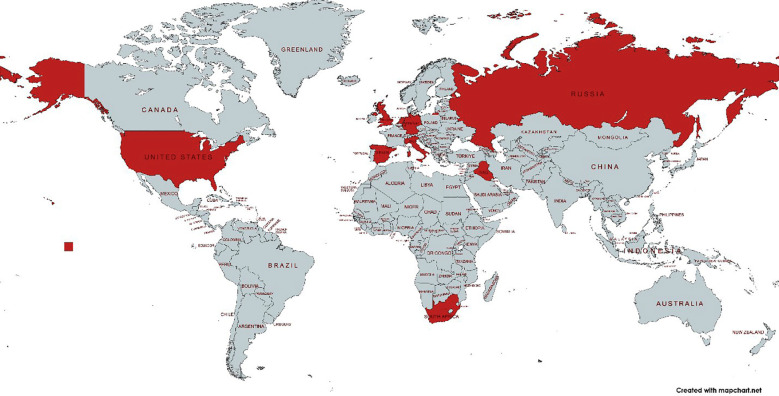
Countries Represented at ICHR-25 Through Speaker and Delegate Participation.

ICHR transformed from being a regional gathering to an internationally renowned forum. In 2025, ICHR featured renowned international experts including Prof. Guido Fumagalli (Italy), Prof. Ana Malashicheva (Russia), Prof. Dr. Saeed Farooq (UK), Prof. Dr. Marti Manyalich (Spain), Dr. Giulio Innamorati (Italy), and Dr. Quiwei Abdullah Pan (Netherlands), all with an H-index greater than 20. In addition, many other distinguished national and international faculty and speakers participated, contributing to the event’s academic depth and global relevance, enriching the intellectual climate, and catalyzing international collaborations, as shown in Fig.2. The inclusion of diverse global experts not only enhanced the academic rigor of the conference but also promoted meaningful dialogues across geographical, cultural, and disciplinary boundaries. It helped align local health research priorities with international benchmarks and encouraged bilateral research partnerships. Cross-border dialogues fostered partnerships and raised awareness of local issues in global contexts.^8^

### Capacity building through pre-conference workshops:

ICHR has increasingly emphasized skill development.^9^

- **ICHR-23:** 10 introductory research workshops
- **ICHR-24:** 31 workshops spanning research methods, dissertation writing, synopsis writing, biostatistics and ethics.
- **ICHR-25:** 52 hands-on sessions covering AI in health research, systematic reviews, mixed methods, academic writing, and grant development.

These efforts trained over 1,000 participants in 2025 alone, offering a highly practical and hands–on learning experience that bridged theory with application. The progressive increase in the number and diversity of workshops is a direct reflection of ICHR’s responsiveness to emerging trends in global health research. This evolution aligns with capacity-building models in LMICs.^10^

Topics such as artificial intelligence, digital tools for data visualization, and academic publishing were not only timely but also in high demand. The involvement of both national and international facilitators ensured quality mentorship and introduced participants to diverse research cultures and perspectives. Furthermore, the workshops acted as entry points for students and early-career professionals to engage in the research ecosystem, with many attendees reporting enhanced confidence in initiating their own studies post-conference. By institutionalizing structured pre-conference capacity-building programs, ICHR successfully bridged the gap between academic theory and practical application, setting a new standard for research-centered events in the region.

### Capacity building through pre-conference workshops:

What sets ICHR apart is its constant evolution:

- **Science Café:** Facilitated open, real-time idea exchange in an informal, intellectually stimulating environment that encouraged interdisciplinary dialogue.^11^
- **Young Investigator Awards:** Encouraged student-led research and recognized early-career contributions, creating a motivating platform for emerging scholars.
- **Digital Transformation:** AI-powered sessions, ChatGPT tutorials, infographics, and data visualization tools empowered participants with future-ready research competencies.
- **CHR Graduation Integration:** Bridged formal research training with real-world academic exposure, symbolizing the continuum of learning and application.
- **Virtual & Hybrid Access:** Enabled global participation, making the event more inclusive and accessible to a geographically diverse audience.
- **Research Ambassador Assembly:** Established a network of student and early-career researchers to promote peer-led engagement and extend impact beyond the conference.

Compared to traditional conference models, ICHR employed flipped and participatory pedagogies that align with best practices in medical education.^12^ These innovations have redefined the academic conference experience in Pakistan by elevating the standard for engagement, accessibility, and impact. ICHR successfully broke away from conventional conference models by embedding dynamic, forward-thinking elements that appealed to a new generation of researchers. This progressive approach not only enriched the educational environment but also positioned ICHR as a pioneer in academic conferencing within the region.

### Institutional capacity and national visibility:

- Beyond the conference itself, ICHR has triggered a ripple effect:
- Elevated RMI as a national research training leader, recognized for embedding research into the academic fabric of its healthcare institutions
- Encouraged multi-institutional research collaborations, fostering a spirit of academic solidarity and shared scientific goals across Pakistan
- Contributed to national policy dialogues on healthcare research by providing a credible platform for evidence-based discussions and stakeholder engagement
- Inspired replication of the ICHR model in other academic institutions across Pakistan, where regional universities have begun organizing their own research conferences and workshops modeled after ICHR’s structure and success

Recent evidence shows that when maintained for several years, these platforms promote research ecosystem reform.^13^ By normalizing research as a vital part of healthcare education, ICHR’s influence has been crucial in encouraging institutions to reevaluate their curricular priorities and make faculty development investments. In addition to raising RMI’s institutional reputation, its leadership in this movement has improved national perceptions of how research influences healthcare outcomes.

## DISCUSSION

The current study explains the impact and evolution of the International Conference on Health Research (ICHR) from 2023 to 2025, focusing on its growth in participation, internationalization, and contribution to institutional research capacity building. The findings of this study suggests that constant academic conferences can strengthen the research ecosystem in low- and middle-income countries such as Pakistan.

A 233% increase in participation, from 600 participants in 2023 to 2,000 in 2025, indicates an increased awareness and institutional commitment toward health research. This increase is similar in trends seen in reports from other universities of Pakistan that have successfully organized institutional research conferences to collaborate among health care professionals.^14^ Moreover similar increase in trends have been observed in other regional academic conferences such as in in India and Bangladesh, where hybrid modes and reduced registration barriers increased the participation.^15^,^16^ The hybrid mode of ICHR likely contributed to this increased number of participation, promotion, inclusivity and accessibility.

The surge in international participation, representation from around 10 countries demonstrates Pakistan’s growing visibility in global health research ecosystem. Similar initiatives comparable to ICHR such as the Sri Lankan Medical Association Congress have reported similar benefits in enhancing and expanding the academic networking and cross border collaborations and contributions.^17^,^18^ The presence of foreign speakers added depth to scientific networking, exchange of ideas, and exposed local participants to diverse methodologies, increasing the quality and potential for future Collaborations.

The number of Workshops increased from 10 in 2023 to 52 in 2025, which provided knowledge and access to over 1,000 participants in the most recent conference. This significant growth demonstrated ICHR’s evolution from a typical scientific meeting to a structured training forum. Comparable short courses and relatable workshops such as the Pakistan Health Research Council’s short courses and international initiatives by the African Academy of Sciences have shown that advanced and skills-based workshops can significantly improve research competency and publication outcomes.^19^,^21^ These capacity-building workshops and short courses covers up the gap between academic interest and practical skill acquisition that is a crucial step in strengthening Pakistan’s health research ecosystem.

One of the highly significant impacts of ICHR has been the drastic rise in enrollment for the Certificate in Health Research (CHR) program. Almost More than 250 new registrations were received in the month surrounding ICHR-25 alone. This increase highlights the catalytic influence of the conference in motivating healthcare professionals in continuing the structured research education. Comparable trends have been reported globally, where academic conferences have increased enrollment in post-conference training and degree programs.^22^

This consistent growth of ICHR also introduced new challenges such as logistical and operational, which included the coordination of international speakers, the modes of delivery, and the event management. These are challenges were faced globally in conferences and courses transitioning to hybrid formats post-COVID-19 The adaptive strategies of ICHR organizing teams such as strategic volunteer management, digital submission portals and real-time feedback mechanisms that align with best practices which is recommended for sustainable academic events.

This study significantly contributes to new evidence in increasing the literature on academic capacity building in LMICs. It also demonstrates how a locally initiated and managed conference can be modified and develop into an internationally recognized event that stimulates the structured research awareness and institutional reform. Beyond the analytics, ICHR has promoted an ecosystem shift in how research is transferred, disseminated, taught, valued, and practiced within Pakistan’s healthcare education system.

### Strengths & Limitations:

One of the major strengths of this study is the utilization of structured institutional data that included the registration records, workshop records, and feedback surveys providing a comprehensive view of ICHR’s multidimensional impact. Moreover, after comparing the conferences of three consecutive years, it captured temporal trends and programmatic evolution. However, the limitations of this study include its descriptive, retrospective design and self-reported feedback, which may introduce response bias. Future studies are suggested to incorporate bibliometric tracking to assess long-term effects on publication output and research productivity.

## CONCLUSION

The trajectory of ICHR from 2023 to 2025 is evidence of the transformational potential of academic dedication, teamwork, and vision. What started out as a regional initiative to support health research has grown into a premier national event acknowledged for its superiority in academic involvement, creativity, and global connections.

In addition to elevating Rehman Medical Institute as a center of academic excellence, ICHR has created a replicable model for other institutions in Pakistan and abroad by focusing strong capacity-building, innovative themes, and dynamic formats. Increased national dialogue on health policy, broader academic networks, and better research literacy are all examples of its knock-on outcomes.

In the future, ICHR is in a strong position to increase its influence internationally. As it develops, its potential to promote institutional transformation, stimulate regional health innovation, and impact the global research conversation becomes more apparent. The initiative is a dynamic catalyst for changing the research culture in Pakistan and worldwide, not just an academic event. The intended expansion of ICHR into the South Asian Consortium on Health Research (SACHR) is one of the most ambitious future developments. Connecting regional academic institutions, fostering international collaboration, encouraging collaborative research projects, and creating a common framework for contextual innovation are the objectives of this consortium. RMI hopes to expand its success to a worldwide scale through SACHR, enhancing the region’s collective voice in international health research.

By continuing to invest in partnerships, capacity-building, and digital innovation, ICHR and its successors will not only sustain momentum but also pave the way for a more integrated and responsive health research ecosystem across South Asia. ICHR is not merely a conference; it is a movement catalyzing academic reform, research literacy, and collaborative innovation.

### Declaration of AI Assistance:

This manuscript was reviewed with the assistance of an AI-based language model, which was used solely for grammar refinement, clarity enhancement, and final proofreading. All content, interpretations, and conclusions are the authors’ own, and the authors take full responsibility for the accuracy and integrity of the work.

### Authors Contribution:

**NA** conceived, designed and did statistical analysis & editing of manuscript, is responsible for integrity of research.

**NA, NA, GR** did data collection and manuscript writing.

**MR** did review and final approval of manuscript.
